# Resting-state EEG measures cognitive impairment in Parkinson’s disease

**DOI:** 10.1038/s41531-023-00602-0

**Published:** 2024-01-03

**Authors:** Md Fahim Anjum, Arturo I. Espinoza, Rachel C. Cole, Arun Singh, Patrick May, Ergun Y. Uc, Soura Dasgupta, Nandakumar S. Narayanan

**Affiliations:** 1 Department of Neurology, University of California San Francisco, San Francisco, CA 94143 USA; 2 Department of Neurology, The University of Iowa, Iowa city, IA 52240 USA; 3 Division of Basic Biomedical Sciences, Sanford School of Medicine, University of South Dakota, South Dakota, SD 57069 USA; 4 Department of Electrical and Computer Engineering, The University of Iowa, Iowa city, IA 52240 USA; 5 Neurology Service, Iowa City VA Medical Center, Iowa city, IA 52240 USA

**Keywords:** Parkinson's disease, Diagnostic markers

## Abstract

Cognitive dysfunction is common in Parkinson’s disease (PD). We developed and evaluated an EEG-based biomarker to index cognitive functions in PD from a few minutes of resting-state EEG. We hypothesized that synchronous changes in EEG across the power spectrum can measure cognition. We optimized a data-driven algorithm to efficiently capture these changes and index cognitive function in 100 PD and 49 control participants. We compared our EEG-based cognitive index with the Montreal cognitive assessment (MoCA) and cognitive tests across different domains from National Institutes of Health (NIH) Toolbox using cross-validations, regression models, and randomization tests. Finally, we externally validated our approach on 32 PD participants. We observed cognition-related changes in EEG over multiple spectral rhythms. Utilizing only 8 best-performing electrodes, our proposed index strongly correlated with cognition (MoCA: rho = 0.68, *p* value < 0.001; NIH-Toolbox cognitive tests: rho ≥ 0.56, *p* value < 0.001) outperforming traditional spectral markers (rho = −0.30–0.37). The index showed a strong fit in regression models (*R*^2^ = 0.46) with MoCA, yielded 80% accuracy in detecting cognitive impairment, and was effective in both PD and control participants. Notably, our approach was equally effective (rho = 0.68, *p* value < 0.001; MoCA) in out-of-sample testing. In summary, we introduced a computationally efficient data-driven approach for cross-domain cognition indexing using fewer than 10 EEG electrodes, potentially compatible with dynamic therapies like closed-loop neurostimulation. These results will inform next-generation neurophysiological biomarkers for monitoring cognition in PD and other neurological diseases.

## Introduction

Cognitive dysfunction is a major non-motor symptom of Parkinson’s disease (PD), affecting ~20% of the individuals with PD at initial diagnosis and leading to dementia in >80% of the individuals with disease progression and aging^1–4^. Because PD-related cognitive symptoms can predict morbidity and mortality, early diagnosis may help counsel families and guide crucial treatment decisions. Detailed neuropsychological testing^5^ is the gold standard for determining cognitive function. However, neuropsychological testing requires several hours and trained examiners and is subject to learning effects, especially with frequent testing^5,6^. Therefore, it is not readily compatible with repeated measurements throughout the day or even within short time intervals such as days or weeks. These features limit its role in capturing cognitive fluctuations in PD or real-time feedback for neuromodulation therapies where cognition can be negatively affected while improving motor function^7–9^. Short screening tests such as Montreal Cognitive Assessment (MoCA)^10^ or computerized batteries such as National Institutes of Health Toolbox Cognitive Battery^11^ (NIH-Toolbox) have similar limitations. MoCA is a high-performing screening tool for detecting PD-related cognitive impairment^12–15^. Computer-based cognitive tests provided by the NIH-Toolbox are also commonly used as standardized measures of cognition for insights into different cognitive domains^16^. While these cognitive measures can be readily measured, they depend on paper or digital text input formats limited by the patient’s motor and language proficiency, as well as by trained examiners during data collection and scoring^17^.

EEG provides a widely available, low-cost measure of cortical neurophysiology which can provide predictive electrophysiological markers for monitoring instantaneous and long-term cognitive functioning^18,19^. EEG-based indexing of PD-related cognitive functions can generate objective, rapid, and robust measures of cognition with continuous measurements in complex environments. More importantly, it can contribute to PD treatments such as closed-loop neurostimulation for avoiding cognitive side effects and improving cognitive symptoms^9,20^. In previous studies, EEG-based analysis has facilitated a more accurate characterization of granular fluctuations and it is sensitive to the abnormalities in cortical function that precede the occurrence of overt disease manifestations^21,22^. EEG recording procedures are generally well-tolerated by individuals with cognitive impairments^21^. Thus, EEG-based cognitive assessment that does not require expert administration and is compatible with repeated or continuous measurement would be of great benefit. Changes in EEG activities correlate with cognitive measures such as MoCA and cognitive tests from NIH-Toolbox in various medical conditions^18,19,23^. However, to date, few EEG-based biomarkers have been found in PD patients that correlate strongly with cognitive performances measured by these tests even though such EEG-based measures can achieve superior assessment of cognitive fluctuations^21,23–25^.

While changes in EEG over multiple spectral rhythms, power ratios, and phase-couplings can correlate with cognition and achieve moderate correlation with several psychometric tests^22,26–30^, most prior EEG-based approaches have focused on specific spectral ranges leading to a limited capturing of EEG activities related to cognition. We recently developed a data-driven approach termed Linear predictive coding EEG Algorithm for Parkinson’s Disease (LEAPD) that can efficiently capture spectral EEG profiles over a broad frequency range using a few parameters and reliably detect PD and PD-related depression^7,31,32^. This technique harnesses the compression power of linear predictive coding (LPC) to capture the shape of EEG power spectra by holistically encapsulating distinctive spectral features and can separate one neurological condition from another. In this study, instead of just binary classifications we utilized and modified LEAPD to develop an EEG-based biomarker that correlates well with several well-established cognitive indices by capturing EEG spectral changes related to cognition. We hypothesized that cognition-related changes in EEG activities occur in multiple spectral rhythms across the power spectrum in PD and efficient capturing of these changes can index cognitive functions. Our goal was to develop a cognitive assessment tool based on neurophysiological changes in cortical activities as observed by EEG. This tool would be rapid, independent of any specific language, and trained expertize while leading to a better understanding of the neurophysiology of cognitive impairment in PD.

In this study, we collected resting-state EEG from 149 participants, conducted traditional spectral analyses, and optimized LEAPD to estimate general cognitive function as measured by the MoCA and cognitive tests from NIH-Toolbox. Our spectral analyses revealed synchronous changes related to cognitive measures over a broad spectrum in EEG. We externally validated the performance of our approach on a separate out-of-sample dataset. Our results will contribute to EEG-based diagnostic technologies for detecting cognitive outcomes quickly and accurately, real-time feedback on the cognitive effects of medical and surgical interventions, and long-term monitoring of cognitive function in PD and other neurodegenerative diseases.

## Results

**Fig. 1:**
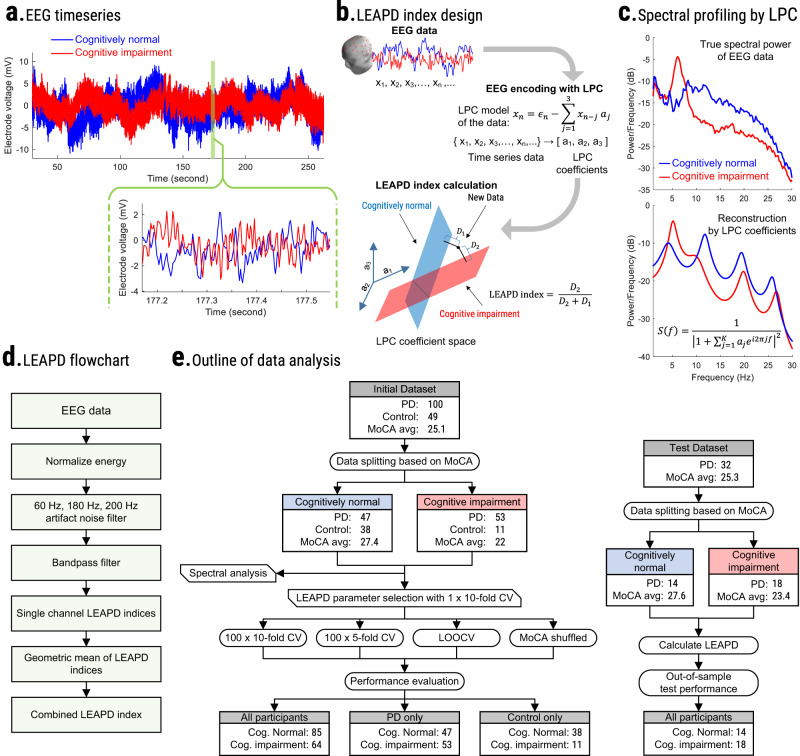
Methodology of the study. **a** Example of EEG time series comparison between a PD participant with normal cognition (blue) and a participant with cognitive impairment (red) from a representative electrode P8. **b** Example illustration of single-electrode LEAPD index: EEG data (${x}_{1},{x}_{2},\ldots ,{x}_{n},\ldots$) are encoded by linear predictive coding (LPC) which fits the data into a 3rd order autoregressive model where each sample (${x}_{n}$) is modeled by the weighted sum of 3 past consecutive samples (middle). These weights (${a}_{1},{a}_{2},{a}_{3}$) are LPC coefficients that represent the EEG data and become a single point in a high-dimensional LPC coefficient space (bottom; LPC coefficients as axes). After finding separate affine subspaces for cognitively impaired (red) and cognitively normal (blue) participants, LEAPD is calculated for new data by encoding it in that space and finding the relative distances from these affine subspaces. ${D}_{1}$ and ${D}_{2}$ are the distances from the new data point to the cognitively normal and cognitively impaired affine subspace respectively. **c** Illustration of spectral profiling via LPC: true spectral power (top) of representative EEG data (P8) from a cognitively normal (blue) and cognitively impaired participant (red). These power spectra show changes in theta, alpha, and beta rhythms related to cognition which are captured by LPC shown by the reconstruction of spectral power from LPC coefficients (bottom). EEG data were bandpass filtered (2–29 Hz). **d** Steps for combined LEAPD index generation from EEG data from multiple electrodes. **e** Schematic and data analysis outline of the study for LEAPD and traditional EEG spectral analysis with randomization test and cross-validations for MoCA (left) using 149 participants (Table 1) and outline of out-of-sample validation test (right) using a separate test dataset of 32 PD participants (Table 1). PD Parkinson’s disease. LOOCV leave-one-out cross-validation. MoCA Montreal Cognitive Assessment.

We recruited 100 PD participants from the Movement Disorders Clinic at the University of Iowa and 49 demographically-matched controls from the general Iowa City community (Table 1). To further assess our findings, we added 32 PD participants from the Aerobic Exercise in Parkinson’s Disease study (NCT03808675) as an out-of-sample test group (Table 1). Resting-state EEG was collected for a few minutes during ON dopaminergic medications (Fig. 1a). To measure cognitive function, we employed a modified LEAPD index (Fig. 1b–d; for details, see Methods) and utilized several cross-validation schemes, randomization tests, and external out-of-sample assessments to evaluate the performance (Fig. 1e).

**Table 1:** Participant demographics.

	Dataset of 149 participants				Out-of-sample test dataset of 32 PD		
	PD-based population group		MoCA-based Classification of cognitive impairment for LEAPD		PD-based group	MoCA-based classification of cognitive impairment for LEAPD	
Condition	PD	Control	Cognitively impaired	Cognitively normal	PD	Cognitively impaired	Cognitively normal
Total	100	49	64	85	32	18	14
Sex	68 M 32 F	26 M 23 F	48 M 16 F	46 M 39 F	21 M 11 F	16 M 2 F	5 M 9 F
PD	100 PD -	- 49 Controls	53 PD 11 Controls	47 PD 38 Controls	32 PD	18 PD	14 PD
Age	68.53 ± 8.06*	70.91 ± 7.62*	71.68 ± 7.8***	67.53 ± 7.66***	67.67 ± 7.91	69.67 ± 7.28	65.07 ± 8.20
MoCA	24.31 ± 4.02***	26.67 ± 1.86***	22 ± 3.49***	27.41 ± 1.18***	25.28 ± 2.45	23.44 ± 1.15***	27.64 ± 1.39***
UPDRS	12.47 ± 7.17	-	13.57 ± 7.46^3^	11.23 ± 6.69^3^	13.16 ± 8.22	15 ± 7.84	10.79 ± 8.36
Dx	4.64 ± 3.79	-	4.53 ± 3.56^3^	4.77 ± 4.08^3^	3.84 ± 3.50	4.12 ± 3.47	3.48 ± 2.44
EEG rec. (min)	2.60 ± 0.75	2.83 ± 0.78	2.7 ± 0.86	2.65 ± 0.69	2.32 ± 0.39	2.15 ± 0.21*	2.54 ± 0.45*
Year of Ed.	15.61 ± 3.00*	16.53 ± 2.12*	15.52 ± 2.68	16.21 ± 2.8	16.56 ± 2.91	16.9 ± 3.25	16.14 ± 2.44
LEDD (mg)	822.47 ± 446.22	-	863.40 ± 507.67^3^	775.33 ± 362.70^3^	543 ± 370.42	593.67 ± 406.84	477.86 ± 320.36
NIH PVT^1^	52.88 ± 07.13**	57.02 ± 07.48**	51.24 ± 07.42***	56.63 ± 06.65***	-	-	-
NIH PCPST^1^	36.45 ± 14.35***	52.67 ± 12.82***	35.03 ± 15.76***	47.14 ± 13.70***	-	-	-
NIH DCCST^2^	50.70 ± 11.91**	57.83 ± 12.66**	50.10 ± 13.00**	55.37 ± 11.81**	-	-	-
NIH FICAT^2^	40.91 ± 09.95***	50.41 ± 09.22***	40.16 ± 09.86***	47.10 ± 10.34***	-	-	-
NIH PSMT^2^	46.62 ± 10.93***	56.52 ± 10.99***	45.97 ± 10.89***	52.97 ± 11.75***	-	-	-

Participant demographics (mean ± standard deviation) for Iowa dataset with a total of 149 participants and the out-of-sample test dataset of 32 PD participants. Wilcoxon non-parametric rank-sum test was used for group-level comparisons. *PD* Parkinson’s disease, *UPDRS* United Parkinson’s Disease Rating Scale (motor), *Dx* years since PD diagnosis, *MoCA* Montreal Cognitive Assessment, *LEDD* L-Dopa equivalent daily dose in mg, *EEG rec.* length of EEG, *Year of Ed.* years of education, *NIH* National Institutes of Health, *PVT* picture vocabulary test, *FICAT* Flanker inhibitory control and attention test, *DCCST* dimensional change card sorting test, *PCPST* pattern comparison processing speed test, PSMT picture sequence memory test. Group-level rank-sum test *p* values: <0.001***, <0.01**, and <0.05*.

^1^Data available for 94 PD subjects and 46 controls.

^2^Data available for 93 PD subjects and 46 controls. Participants were classified into cognitively impaired and cognitively normal groups based on MoCA scores.

^3^No available data for controls for UPDRS, LEDD, and years since PD diagnosis and only PD values were used for statistical measures.

### Correlation of EEG spectral features and LEAPD with cognitive measures

**Fig. 2:**
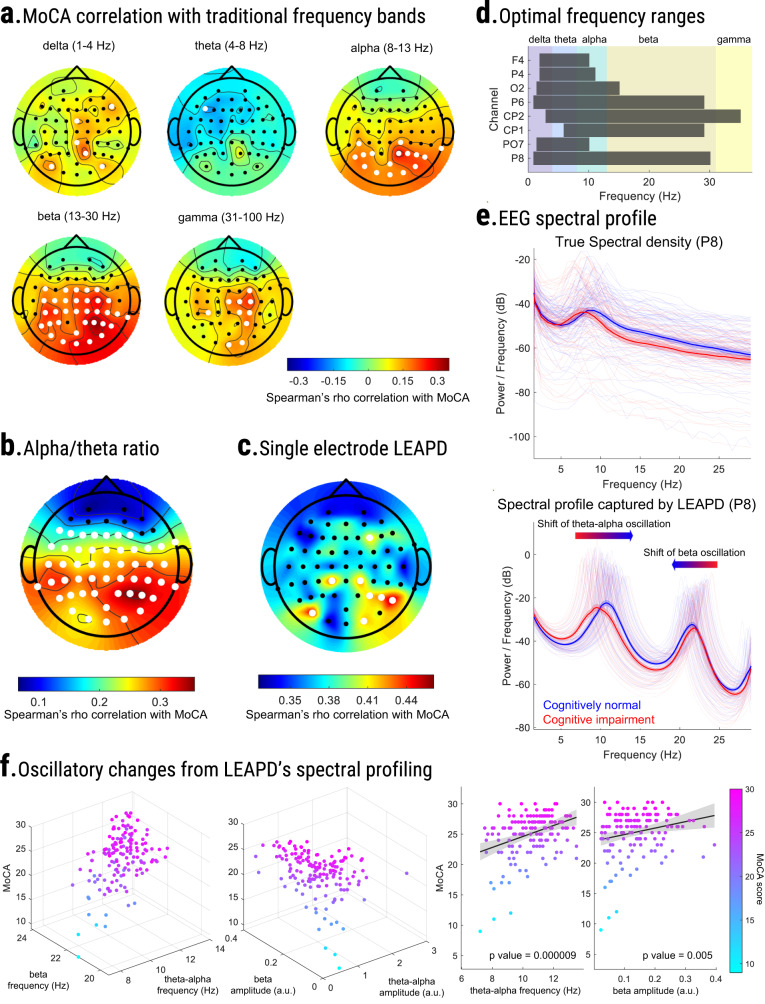
EEG feature analysis and parameter choice. Topographic plots of age-adjusted Spearman’s rho correlation between MoCA scores and **a** traditional frequency bands, **b** Log-spectral ratio of alpha (8–13 Hz) and theta (4–8 Hz) rhythm. Electrodes marked as white signifies a statistically significant correlation (*p* value < 0.05). **c** Topographic plot of correlations between single-electrode LEAPD indices and MoCA scores during parameter selection for combined LEAPD index using 10-fold single-round cross-validation with the 149 participants (Table 1). Selected electrodes are marked as white. These were utilized across all participants in performance evaluations and analyses except the robustness performance of LEAPD. **d** Optimal frequency ranges that resulted in the maximum correlation between MoCA and LEAPD in a single-round 10-fold cross-validation during the parameter optimization of LEAPD at selected EEG electrodes. Vertical color blocks represent canonical frequency bands. **e** Comparison of spectral densities between cognitively impaired (red) and cognitively normal (blue) groups from single-electrode (P8) raw EEG data (top) and EEG-encoded linear predictive coding (LPC) models (bottom) capturing unique spectral profiles with dominant oscillations in theta-alpha (7–13 Hz) and beta rhythms. EEG data were bandpass filtered (2–29 Hz) before the encoding and LPC order was 7. Thick lines show mean spectral densities; lighter lines are individual spectral densities, and shaded areas show the standard error of the mean. The arrows mark the directions of the shifts of the spectral peaks from cognitive impairment to normal cognition. **f** Frequencies and amplitudes of the oscillatory modes captured by LPC in panel e vary with MoCA scores in a statistically significant manner: 3D scatter plots (left) of theta-alpha (7−13 Hz) and beta oscillations (13–25 Hz) captured by LPC with MoCA scores (z axes) in oscillation frequency (Hz) and amplitude (a.u.). Each dot represents data from one participant and is colored according to MoCA (color bar; right). The 2D scatter plots (right) show linear regression models where theta-alpha oscillation frequency and beta oscillation amplitude increase with MoCA scores. Data in all panels are from 149 participants (Table 1).

First, using the EEG data of 149 participants (Table 1), we correlated LEAPD and traditional EEG spectral power features with MoCA score, a screening tool for cognition^5,33^. Spectral analyses of EEG revealed moderate correlations with MoCA scores (Fig. 2a, b). In particular, MoCA score showed statistically significant correlations with increased beta (13–30 Hz) power in central-parietal region (highest at P4: rho = 0.37, *p* value < 0.001, Fig. 2a), increased alpha (8–13 Hz) power in parietal region (highest at P2: rho = 0.29, *p* value < 0.001), increased gamma (31–100 Hz) power in central-parietal region (highest at C4: rho = 0.25, *p* value = 0.002), increased delta (1–4 Hz) power in central-parietal region (highest at P2: rho = 0.25, *p* value = 0.002) and reduced theta (4–8 Hz) power in left frontal region (F5: rho = −0.17, *p* value = 0.04). For investigating the correlation between spectral ratios and MoCA scores, we chose the alpha/theta log-spectral ratio^30^ which simultaneously captures reduced theta and increased alpha power. The alpha/theta ratio showed a statistically significant correlation in almost all regions with MoCA (highest at P4: rho = 0.36 *p* value < 0.001, Fig. 2b). Furthermore, the highest correlation in beta power and alpha/theta ratio were both in the parietal region (P4) implicating the possibility of achieving high correlations with holistic approaches that can capture changes in EEG activities over a broad spectrum.

Next, we tested the hypothesis that LEAPD, which captures the spectral profile of EEG over a broad range, can measure cognitive function in PD. Individual electrode performances on the data of 149 participants (Table 1) demonstrated that LEAPD indices from single electrodes strongly correlated with MoCA score (Fig. 2c; Spearman’s rho > 0.41 noted for P8, PO7, CP1, CP2, P6, O2, P4, and F4). This information was useful in determining which electrodes might be most informative for indexing cognition in PD. We selected these eight top-performing electrodes to calculate a combined LEAPD index across all participants. We also used the same eight electrodes during the out-of-sample test. The best-performing LEAPD frequency ranges for the central-parietal electrodes (P6, CP1, CP2, and P8) were broad while PO7, O2, P4, and F4 were focused on lower frequencies (Fig. 2d). Differences in EEG spectral densities on a broad spectrum were evident in the data-driven central-parietal electrodes between cognitively impaired and cognitively normal participants, regardless of whether they had PD which was consistent with our spectral power analyses (Fig. 2e). These differences were captured by the EEG spectral profiles of LPC models through the shift of oscillations in theta-alpha (7–13 Hz) and beta oscillations (13–25 Hz; Fig. 2e, f). The frequency and amplitude of oscillatory modes captured by LPC showed statistical significance in explaining MoCA scores in linear regression models where theta-alpha oscillation frequency and beta oscillation amplitude increased with MoCA scores (Fig. 2f, MoCA vs theta-alpha oscillation frequency: $\beta$ = 0.9, *p* value = 0.000009, MoCA vs beta oscillation amplitude: $\beta$ = 10.64, *p* value = 0.005). These were consistent with the findings in our spectral power analyses where we observed high MoCA correlations with increased beta power and alpha/theta ratio (Fig. 2a, b). Thus, the LPC spectral profiling illustrated the effectiveness of LEAPD for capturing spectral changes in canonical brain rhythms.

Correlations between MoCA score and the combined LEAPD index were remarkably high (rho = 0.68, *p* value < 0.001) across all cross-validation schemes (±0.016; Table 2; Supplementary Table 1). MoCA score correlations with LEAPD indices were two times higher and significantly stronger than those from spectral analyses (LEAPD vs. beta power from P4: z = −4.4, *p* value = <0.001; rho = 0.68 vs. 0.37). MOCA and LEAPD index correlations were also twice as high as the highest previously reported MoCA correlation achieved by EEG features in PD^24^. Correlations with MoCA scores were similar (rho = 0.66 ± 0.024) for participants with PD alone, but lower in control participants (rho = 0.46 ± 0.006; Table 2; Supplementary Table 1). For both PD patients and control participants, the relationship between LEAPD index and MoCA score remained reliable when accounting for sex, UPDRS III, disease duration, LEDD, age, and GDS with linear models (PD: F(1,91) = 63.9, *p* value < 4 × 10^−12^, partial *η*^2^ = 0.28, *n* = 100; Control (sex, age, and GDS only): F(1,44) = 25.3, *p* value < 9 × 10^−6^, partial *η*^2^ = 0.34, *n* = 49) or with Spearman partial correlations (PD: rho ≥ 0.69, *p* value < 3 × 10^−15^ for all factors; Control: rho ≥ 0.47, *p* value < 9 × 10^−4^ for sex, age and GDS). During the randomization test where we shuffled MoCA scores among all participants, MoCA correlations obtained by LEAPD were very low and not statistically significant (rho = 0.02, *p* value = 0.77; Supplementary Table 2). In the out-of-sample test dataset (32 PD, Table 1), combined LEAPD index achieved a high correlation with MoCA score (rho = 0.68, *p* value < 0.001, Table 2) providing strong external validation of LEAPD performance. While we presented correlation results using Spearman’s rho, we observed similar results with Pearson correlation. However, the bounded non-gaussian feature of LEAPD scores made Spearman’s rho a more robust measure of correlations compared to Pearson’s^34,35^.

**Table 2:** Performance summary of LEAPD.

		Leave-one-out cross-validation^1^ (*n* = 149)			Out-of-sample test^2^ (*n* = 32)
		All 149 participants	100 PD only	49 Controls only	All participants (32 PD)
Spearman’s-rho correlation with cognitive measures	MoCA	0.70***	0.69***	0.47***	0.68***
	NIH PVT	0.66***	0.71***	0.55***	-
	NIH PCPST	0.59***	0.57***	0.15	-
	NIH DCCST	0.70***	0.72***	0.68***	-
	NIH FICAT	0.62***	0.61***	0.48***	-
	NIH PSMT	0.57***	0.55***	0.02	-
Linear regression model for MoCA	${R}^{2}$	0.40	0.37	0.34	0.46
	RMSE	0.12	0.12	0.11	0.09
	F-statistic	99.07***	58.79***	24.60***	25.79***
	AIC	−209.14	−135.38	−77.79	−57.29
Quadratic regression model for MoCA	${R}^{2}$	0.48	0.47	0.38	0.46
	RMSE	0.11	0.11	0.10	0.09
	F-statistic	66.06***	42.49***	14.23***	12.49***
	AIC	−226.40	−149.31	−78.76	−55.32
Detection of MoCA-based cognitive impairment	Accuracy %	79.87	79.00	81.63	71.88
	AUC %	0.91	0.91	0.88	0.92
	Sensitivity %	79.69	83.02	63.64	50
	Specificity %	80.00	74.47	86.84	100
	PPV %	75.00	78.57	58.33	100
	NPV %	83.95	79.55	89.19	60.87
	Odds ratio	15.30	13.77	10.73	-

*PD* Parkinson’s disease, *RMSE* root-mean squared error, *AUC* Area under the receiver operating characteristic curve, *AIC* Akaike information criterion, *PPV* positive predictive value, *NPV* negative predictive value, *MoCA* Montreal Cognitive Assessment, *NIH* National Institutes of Health, *PVT* Picture vocabulary test, *FICAT* Flanker inhibitory control and attention test, *DCCST* dimensional change card sorting test, *PCPST* pattern comparison processing speed test, *PSMT* picture sequence memory test. For the regression models, *p* values show the statistical significance of the predictor variable (combined LEAPD index). In all cases, *p* value < 0.001***.

^1^using data from 149 participants (Table 1).

^2^using external validation data from 32 Parkinson’s disease participants (Table 1).

**Fig. 3:**
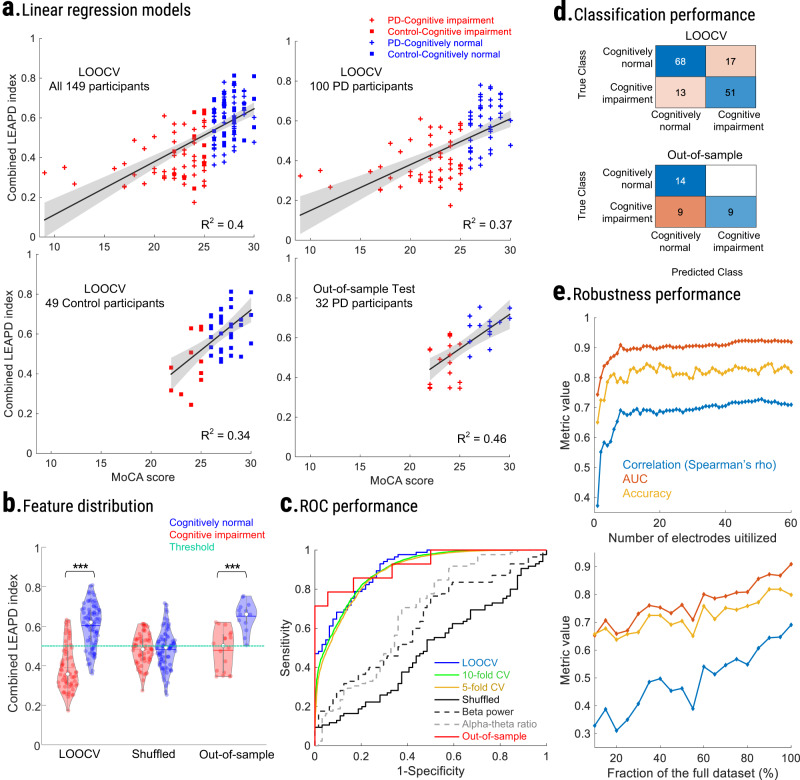
Performance evaluation of LEAPD. **a** Scatter plot for linear regression models between MoCA and combined LEAPD indices for all 149 participants (top left; Table 1), Parkinson’s disease-only (top right; *n* = 100), and controls (bottom left; *n* = 49) during LOOCV and for 32 out-of-sample PD participants (bottom right; Table 1). All models were statistically significant (Table 2). **b** Violin plots of combined LEAPD indices (*y* axis) for cognitively impaired (red) and cognitively normal (blue) participants during LOOCV with 149 participants (Table 1) before (left) and after MoCA score shuffling among participants (middle) and during out-of-sample test with 32 PD (right). The green dotted line represents the detection cutoff (value = 0.5). In all cases, *** indicates group-level rank-sum test *p* value < 0.001. **c** Receiver operative characteristic (ROC) curve for LEAPD in various cross-validations as well as in shuffled MoCA data (solid black), and for the top-performing traditional spectral features (dashed lines; beta power and alpha/theta log ratio at P4) in data from 149 participants. In addition, ROC performance in out-of-sample test with 32 PD was compared (red). **d** Classification performances of LEAPD with confusion matrices for 149 participants during LOOCV (top) and 32 PD participants (bottom) during out-of-sample test. **e** Robustn**e**ss of LEAPD performance: Spearman’s rho correlation with MoCA scores (blue), classifier accuracy (yellow), and AUC (red) performance of LEAPD while varying the number of EEG electrodes utilized for the combined LEAPD index (top). The *x* axis is the number of EEG electrodes utilized, and the *y* axis is the metric (AUC, classifier accuracy, or Spearman’s rho value). LEAPD performance in terms of correlation with MoCA (blue), classifier accuracy (yellow), and AUC (red) while truncating the dataset (bottom). The *x* axis is the size of the dataset after truncation compared to the original size in percentage. Data in panel e from 149 participants (Table 1). PD Parkinson’s disease, LOOCV leave-one-out cross-validation, CV cross-validation.

To further explore the relationship between the combined LEAPD indices and MoCA scores, we used regression analyses. Both linear and quadratic regression models for the combined LEAPD index and MoCA scores were statistically significant (linear and quadratic *p* values < 0.001; Fig. 3a; Table 2; Supplementary Table 1). In the cross-validation schemes conducted with 149 participants, *R*^2^ increased by 7% from linear to quadratic regression (quadratic: 0.46 ± 0.018; linear: 0.39 ± 0.016; Table 2, Supplementary Table 1), and the quadratic model provided significantly stronger fit compared to the linear model (likelihood ratio test with leave-one-out cross-validation; *p* value < 0.001). Regression models achieved higher *R*^2^ for PD participants compared to control participants (Table 2). No statistically significant effect of PD was observed after including it in the linear model as a random effect (mixed-effect vs. no-effect: *p* value = 0.35; Fig. 3a). The same conclusion was obtained for quadratic regression (mixed-effect vs. no-effect: *p* value = 0.98). Regression models for MoCA-shuffled data were not statistically significant (Supplementary Table 2). In the out-of-sample dataset (32 PD participants) both linear and quadratic regression were also statistically significant (linear and quadratic *p* values < 0.001; Fig. 3a; Table 2). However, quadratic regression did not show any improvement over linear regression (likelihood ratio test; *p* value < 0.86). No additional assumptions were made during the regression analyses. These data suggest a relationship between the combined LEAPD index and MoCA scores that is statistically independent of PD, and that the algorithm might be triggered by aspects of the EEG signals related to cognitive status, irrespective of PD.

We also correlated EEG spectral power in canonical bands with cognitive scores from the NIH-Toolbox (Supplementary Fig. 1). Alpha and beta power correlated with PVT scores in the parietal region with the highest correlation of 0.24 (beta power at CP3, *p* value < 0.005). PCPST, PSMT, and DCCST scores correlated with decreased theta and increased beta power with the highest correlation of −0.29 (theta power at C6, *p* value < 0.001), −0.30 (theta power at T8, *p* value < 0.001), and 0.23 (beta power at PO8, *p* value < 0.001) respectively. Alpha, beta, and gamma power showed significant correlations with FICAT scores with the highest correlation of 0.36 (beta power at P4, *p* value < 0.001). There were variations in the EEG spectral power dynamics and their locations for these cognitive tests which could be due to the specific aspects of cognition measured by these tests (Flanker: measure of inhibitory control, DCCST: cognitive flexibility, PSMT: episodic memory, etc.) as dynamic changes in EEG power spectral during these cognitive tests can be different^23^. In contrast to canonical spectral powers, the combined LEAPD index showed high correlations with the cognitive scores across all cross-validations (rho in PVT: 0.64 ± 0.01, PCPST: 0.58 ± 0.004, DCCST: 0.68 ± 0.02, FICAT: 0.61 ± 0.01, PSMT: 0.56 ± 0.007; Table 2, Supplementary Table 1, Supplementary Fig. 2). No statistically significant correlation was found between LEAPD and NIH-Toolbox cognitive scores in the randomization test for all participants (Supplementary Table 2). Taken together, these data demonstrate that LEAPD index could strongly correlate with cognition in PD.

### Detection of cognitive impairment

For all 149 participants during cross-validations, LEAPD classified cognitive impairment and normal cognition with 0.90 ± 0.01 AUC (area under the receiver operating curve), 80.44 ± 0.55% classifier accuracy, 79.52 ± 0.65% sensitivity, and 81.14 ± 0.99% specificity across all cross-validation schemes (Fig. 3b–d; Table 2; rank-sum test on LEAPD scores for cognitive impairment vs. cognitively normal group: *p* value < 0.001) while the top-performing traditional spectral features yielded moderate AUC (alpha-theta ratio: 0.68; beta power: 0.63; electrode P4; Fig. 3c). In PD participants only, LEAPD performance was similar to the case of all participants (0.90 ± 0.01 AUC, 78.90 ± 0.09% classifier accuracy, 82.62 ± 0.55% sensitivity, and 74.70 ± 0.58% specificity). This classification of PD-related cognitive impairment was stronger than previous studies (AUC was 17.6% higher)^36^. For control participants only, LEAPD demonstrated higher classifier accuracy and specificity (83.59 ± 1.87% classifier accuracy, 89.11 ± 2.03% specificity), but lower AUC and sensitivity (0.85 ± 0.02 AUC, 64.55 ± 1.82% sensitivity). In the randomization test with MOCA-shuffled data, the results were close to chance (50.34% classifier accuracy, 0.49 AUC, *p* value = 0.86; Supplementary Table 2). Finally, we checked the potential effects of the group sizes by subsampling the dataset of 149 participants (Table 1) with equal numbers of cognitively impaired and cognitively normal participants in leave-one-out cross-validation which provided similar performances (0.91 ± 0.02 AUC, 79.70 ± 2.79% classifier accuracy, 79.60 ± 3.50% sensitivity, and 79.80 ± 2.90% specificity; Supplementary Fig. 3) to the cross-validation performance for all 149 participants (Table 2). Finally, in the external validation test with 32 PD participants, LEAPD achieved similar performances in AUC and accuracy (0.92 AUC, 71.88% classifier accuracy; Table 2, Fig. 3b–d) but showed lower sensitivity and higher specificity (50% sensitivity, 100% specificity) compared to the performance in the data of 149 participants. This indicates that potentially a ROC-based classification threshold in LEAPD could perform better in larger datasets.

### LEAPD robustness

To investigate the robustness of LEAPD, we examined how correlation and classification performances vary with the number of electrodes (1–60, Supplementary Video 1; see Methods). The correlation of the combined LEAPD index with MoCA score and the AUC of cognitive classification both reached their respective maximums with 51 electrodes while the maximum classifier accuracy was achieved with 16 electrodes (Fig. 3e). However, with more than 10 electrodes, improvements in correlation, AUC, and classifier accuracy were marginal. In addition, we investigated the robustness of the LEAPD approach by using truncation analysis to quantify the effect of EEG signal length (i.e., duration of EEG data) on performance. During data truncation with 10% of the data, LEAPD achieved an AUC of 0.65 (Avg. EEG length = 16 seconds; rho = 0.33, 65.77% classifier accuracy; Fig. 3e). With 40% of the data, it achieved 0.75 AUC (Avg. EEG length = 1 minute; rho = 0.5, and 72.48% classifier accuracy). The results during data truncation show a gradual increase in performance indicating that the algorithm might benefit from longer EEG recordings. Performances of LEAPD in MoCA-shuffled data (randomization test) during data truncation were not statistically significant (45.57 ± 3.22% classifier accuracy, 0.44 ± 0.03 AUC, rho = −0.07 ± 0.06). Taken together, these results indicate that LEAPD can powerfully identify PD-related cognitive impairment from a limited electrode montage and a few minutes of resting-state EEG.

## Discussion

In this study, we hypothesized that broad-spectrum EEG activity changes with cognition and capturing these changes can lead to EEG-based cognitive measures. Our spectral analyses indicated that EEG activities over multiple spectral rhythms correlate with cognitive measures. We found that LEAPD, a data-driven machine learning algorithm^32^, can efficiently capture EEG spectral profiles for cognition and detect cognitive dysfunction both in PD and control participants. Our data showed that by rapidly encoding resting-state EEG data from eight electrodes, LEAPD can accurately index cognitive function as measured by MoCA or by the cognitive tests from the NIH toolbox. It also detected cognitive impairment in control participants, performed consistently across multiple cross-validation schemes, provided reliable performance during data truncations, and showed no indication of overfitting in the randomization tests. The lack of significant correlation between LEAPD and cognitive tests with randomly shuffled cognitive scores showed the dependency of LEAPD’s performance on capturing the neurophysiological changes related to cognition. Finally, we validated the performance of LEAPD on a separate out-of-sample dataset of 32 PD participants. Thus, LEAPD can be useful for finding efficient EEG-based biomarkers for cognitive impairment in humans including individuals with PD.

Strong correlations of LEAPD-based cognitive indices with PVT, DCCST, PCPST, PSMT, and FICAT scores in addition to MoCA suggest that cognitive impairment involves dysfunction in cortical networks that manifest in scalp EEG^21,37^ and that our approach is sensitive to cognitive function across different domains. Although various other measures have been used to assess cognitive impairment via EEG^37–39^, to the best of our knowledge no other technique has this level of accuracy, correlation in PD patients, and computational efficiency. While LEAPD was useful in both PD and demographically similar control participants, its lower performance with control participants might be due to the limited range of performance in this group with a small number of cognitively impaired participants. At its core, LEAPD is a data-driven method originally designed for detecting PD that captures characteristic spectral EEG changes and has shown strong performances in binary classification between data population groups such as PD vs control, PD with vs without depression, and depression with vs without PD with high accuracy^7,32^. This study is an advance because we were able to modify LEAPD by changing its training sub-groups and parameter selection metric for designing EEG-based indexing of cognition which was highly predictive of cognitive impairment as measured by MoCA and by the NIH-Toolbox. LEAPD was able to provide cognitive markers in both PD and control participants by rapidly capturing the EEG spectral profiles while showing no significant effects of PD in the detection of cognitive impairment. Furthermore, we rigorously validated the performance of our approach using a separate dataset in out-of-sample testing along with randomization and robustness tests. Thus our findings represent highly validated results. The effectiveness of LEAPD indices might lead to early diagnosis of cognitive impairment in not only PD but also other brain diseases that impair cognition that manifests through EEG such as Dementia with Lewy bodies (DLB)^21^.

Although purely data-driven, the best-performing frequency bands revealed by LEAPD showed neurophysiological significance and correlation with cognitive measurements in previous studies. Indeed, LEAPD may identify neurophysiological signatures of cortical function that are linked to cognitive dysfunction. For example, LEAPD spectral bands for MoCA correlation include theta and delta rhythms which are predictive of MoCA scores, have strong correlations with several cognitive measures, and reflect high-level cognitive processes and mechanisms for cognitive control^24,26,29,40^. Furthermore, changes in EEG activities related to cognitive function can occur synchronously over multiple spectral rhythms. In PD, delta-alpha coupling correlates with cognitive measures^26^, and cue-related medial frontal delta-theta powers correlate with cognitive function^24,25,41^. Abnormal delta and alpha rhythms in the posterior brain regions can index the decline of cognitive visuo-spatial function^37^. Furthermore, delta, theta, alpha, and beta activities in EEG and their ratios reflect cognitive function in PD^22,36,41,42^. While these changes in multiple EEG rhythms are traditionally captured with spectral ratios which can correlate with cognitive measures in PD^27,28,30^, these ratios are limited by the predefined frequency ranges of the spectral bands. In contrast, LEAPD is not strictly confined to the canonical rhythms and captures simultaneous changes in multiple EEG frequencies by extracting the spectral profile in a holistic fashion, which leads to an efficient estimation of cognitive dysfunction. Furthermore, LEAPD can reveal changes in the underlying oscillatory modes of the EEG signal by utilizing LPC. Thus, unlike other machine learning approaches where the mechanisms and features are not well understood, LEAPD can provide insight into how markers for cognition are detected and identified. In our study, the dominant oscillations captured by LEAPD at central-parietal electrodes indicated gradual shifts in theta-alpha (7–13 Hz) oscillation frequency and beta oscillation amplitude with cognitive function (Fig. 2e, f). Interestingly, spectral peak shifts of theta-alpha EEG rhythms in posterior cortical regions can also predict dementia development in PD^38,43^. Shifts of theta-alpha peak frequency with cognition, as discovered by LEAPD (Fig. 2f), have also been observed previously in PD^20^. This also explains the high correlation achieved by alpha/theta log-spectral ratio in our study (rho = 0.36, Fig. 2b) as well as in previous studies^30^. Theta rhythms can vary with activity in the default mode network while alpha rhythms can be higher during low arousal resting states and beta synchronization occurs during the execution of cognitive tests^23^. These demonstrated that in addition to spectral power, oscillations represented by LPC and LEAPD can be utilized as a tool for investigating cortical neurophysiology. Future studies will further investigate the oscillatory modes captured by LEAPD over a wider variety of participants with cognitive impairments (due to PD, Alzheimer’s, DLB, etc.) for a better understanding of the underlying neurophysiological mechanisms. Finally, unlike prior studies^23^, we analyzed resting-state EEG activities that were not influenced by the cognitive measurement procedures.

Neuropsychological testing has a key role in cognitive neurology and can provide evidence-based diagnostic and therapeutic guidance^5,44^. However, EEG-based cognitive markers provided by LEAPD might be a useful complement to classical neuropsychological tests in clinical scenarios where such detailed testings are not feasible^45^, and by choosing candidates as a large-scale screening and follow-up tool for further detailed testing leading to a more feasible utilization of such valuable resources and trained health professionals that are sometimes not immediately available. Because LEAPD uses LPC to compress key features of EEG spectra, it is computationally efficient and suitable for real-time applications^31,32^. Indeed, after initialization and LEAPD configuration, LPC vectors can be computed from any new EEG data within seconds^31^. Applying a full montage of EEG electrodes and collecting high-quality resting-state data can take ~20 minutes. However, in our study, LEAPD required eight electrodes and performed reliably on EEG data which were ~3 minutes long on average without any manual artifact cleaning. In our out-of-sample test, these eight EEG electrodes showed robust predictions of cognition on a separate dataset of 32 PD. Thus, our findings may minimize the need for high-density scalp EEG and suggest that LEAPD can be implemented in wearable EEG devices^46^ which can facilitate EEG data acquisition time making it suitable for broader implementations. LEAPD can capture EEG-based cognitive markers within a few minutes and thus could effectively identify the characteristic cognitive fluctuations of DLB or PD dementia^21,38^. LEAPD can complement neuropsychological testing because it is fast, repeatable, and independent from patient-related factors such as fatigue and challenges such as severe motor symptoms or dementia. Despite these data, EEG is not without limitations, and future longitudinal studies are needed to explore these practical aspects in detail. Furthermore, combined with advanced therapies such as adaptive neurostimulation with long-term wireless streaming of neural recordings^47^, EEG-based assessment of cognitive function by LEAPD might help design better feedback signals or select appropriate neurostimulation settings. In addition, LEAPD might mitigate treatment-related or stimulation-related cognitive side effects^9^.

Our study has several limitations. First, scalp EEG has poor spatial resolution whereas MEG, fMRI, or intraoperative recording studies might provide detailed spatial information about brain activity, which could contribute to more accurate cognitive measurements of LEAPD. Techniques such as MRI are also better suited to capture cortical atrophy, which may affect EEG signals contributing to LEAPD’s prediction of cognitive function. Despite the poor spatial resolution, we chose EEG as it is widely available, low-cost, and has shown promising results as a neurophysiological marker compared to others including fMRI, SPECT, PET, and neurophysiological tests in neurological conditions such as DLB^21^. Second, MoCA-based classification (cutoff 26/30) for cognitive impairment may not be as accurate as classification using Level II testing for cognitive impairment in PD^5^. However, our primary goal of this study was to index cognition using EEG that strongly correlates with standard measures of cognition. Third, our study is cross-sectional and represents a moderately sized convenience sample and did not follow patients over time. Longitudinal studies with larger cohorts with repeated measurements are needed to determine the stability of LEAPD over time, the predictive value of our EEG-based markers on cognitive outcomes, and their usefulness in monitoring cognition in PD in clinical practice. Fourth, our study was ON levodopa as usual, although our past work indicates that levodopa does not change EEG spectra, and our previous work showing levodopa differences was in rodent striatal field potentials, which are vastly different than scalp EEG^31,48^. We also did not collect data on the laterality of motor symptoms, as this may play a role in cognition in PD^49,50^. Furthermore, our control participants may not reflect the general population. Lastly, we had a moderate-sized dataset and thus evaluated the performance of LEAPD scores using cross-validation schemes, commonly utilized in measuring diagnostic performances^51,52^. Furthermore, we performed extensive k-fold cross-validations for various k values with repetitions using independent shuffling of the folds at subject-level for proper estimation of model’s performance in a diagnostic scenario^51,52^. Performance variations of LEAPD indices across these cross-validation schemes were ≤2% (Table 2; Supplementary Table 1) and robustness analyses showed stable performance. In addition, we trained LEAPD on data with randomly shuffled cognitive scores and did not find consistent results, suggesting that the correlations with cognitive measures were not achieved through fitting noise or data artifacts. Most importantly, we conducted external validation with an out-of-sample dataset of 32 PD participants which showed robust performance even on an independent and separately acquired out-of-sample test. Despite these points, it is possible that our LEAPD analyses overestimate the sensitivity and specificity from routine EEG, and future studies will scale this work to additional centers and conditions. However, to the best of our knowledge, this is the only study to date where an external dataset was utilized to validate the performance of a cognitive index.

In summary, we found that LEAPD derived from resting-state EEG can capture cognitive changes in PD. This could be useful as a potential marker to infer cognitive impairment in PD as well as other neurodegenerative diseases. Because LEAPD is scalable and amenable to real-time applications, it also might inspire novel feedback-based interventions or advanced neuromodulation therapies targeted at cognition.

## Methods

### Participants and cognitive measures

We recruited 100 PD participants from the Movement Disorders Clinic at the University of Iowa, Iowa City between 2017 and 2022 (Table 1). A movement disorders physician diagnosed each individual with PD according to the United Kingdom Brain Bank criteria. All procedures were performed while participants were taking their usual medications. Additionally, 49 demographically matched controls without known neurological disease were recruited from the general Iowa City community between 2017 and 2022 through the Seniors Together in Aging Research registry (https://icts.uiowa.edu/star). We reported some of these data in our previous studies but focused primarily on motor control, cognitive control, or neuropsychiatric symptoms^24,25,45^. We also recruited 32 new and independent PD participants from the Aerobic Exercise in Parkinson’s Disease (NCT03808675) for our prospective out-of-sample test (Table 1). The motor component of the United Parkinson’s Disease Rating Scale and the MoCA scores were administered by trained raters. Participants also completed other clinical assessments of cognition, mood, and gait. The study was approved by the University of Iowa Institutional Review Board (protocol # 201707828). Written informed consent was provided by all participants. We used MoCA to quantify cognitive condition among participants as it is more sensitive to cognitive deterioration in PD^12–14^. We defined cognitive impairment as MoCA scores < 26 and cognitively normal as MoCA scores 26–30^10,15,33,53^. Among the 149 participants, 53 PD and 11 control participants had MoCA-defined cognitive impairment (MoCA score <26). The control participants with cognitive impairment had no pre-existing neurological or psychiatric disease. The median MoCA score among the 149 participants was 26, and the range for controls was 22–30 while the range for PD participants was 9–30. To assess specific aspects of cognitive functioning, we used NIH-Toolbox, a standardized computer-based neuropsychological screening battery^11^. Five tests from the NIH-Toolbox were administered to the participants: Picture vocabulary test (PVT), Pattern comparison processing speed test (PCPST), Dimensional change card sorting test (DCCST), Flanker inhibitory control and attention test (FICAT), and Picture sequence memory test (PSMT). The tests were administered through iPads and extensive user directions were provided by the examiner. In the out-of-sample dataset, 18 PD participants had MoCA-defined cognitive impairment (range: 22–25), 14 participants had normal cognition (range: 26−30) and the MoCA score (25.3 ± 2.5, *n* = 32) range among all 32 PD participants was 22–30 (Table 1).

The goal of this study was to investigate LEAPD as an index for PD-related cognitive impairment. To modify and optimize LEAPD for MoCA, EEG recordings from the 149 participants were divided into two groups: cognitively impaired participants (MoCA score <26; *n* = 64; 53 PD, 11 control) and cognitively normal participants (MoCA score ≥ 26; *n* = 85; 47 PD; 38 control; Table 1). To optimize training models for each of the cognitive tests from NIH-Toolbox, all participants were similarly divided into two respective training groups based on their scores (median as cutoff) while the classification of normal cognition and cognitive impairment was based on only MoCA scores. We chose medians as cut-offs for constructing two balanced training groups for optimizing LEAPD.

### EEG recordings

Resting-state EEG was collected from participants while they sat in a quiet room with their eyes open to avoid drowsiness and the posterior dominant rhythm. EEG data was recorded for a few minutes ON dopaminergic medications, as usual, using a 64-channel actiCAP and Brain Vision system (Brain Products GmbH) with a 0.1-Hz high pass filter and a sampling frequency of 500 Hz (Fig. 1a). Data from 60 electrodes were used for each participant (Pz was the online reference; Iz, I1, and I2 were inconsistent among the participants and were excluded). EEG time series data from each electrode were normalized to maintain uniform amplitude scaling across all electrodes. This was accomplished by dividing each time series data by the square root of its total energy. Additionally, line noise artifacts at 60 Hz, 180 Hz, and 200 Hz were eliminated in the frequency domain through the application of FFT (Fast Fourier Transform). No further manual inspection, cleaning, or artifact removal was conducted. On average, EEG electrodes could be applied in ~20 minutes, and resting-state data can be acquired in ~10 minutes.

### LEAPD: EEG-based feature for cognition

LEAPD is a data-driven approach originally developed as an efficient EEG-based marker that can reliably detect PD-related changes in preclinical animal models as well as in humans. This approach strongly outperformed prior methods for the detection of PD and could detect depression in PD^7,31,32^. The key advance of LEAPD is its encoding of EEG time series through LPC which has a long history of compression, encoding, and modeling applications in myoelectric signals, configurations of vocal tract, and speech analysis^54,55^. Fundamentally, LPC fits EEG time series data into an autoregressive model where each data sample can be estimated by the weighted sum of the previous samples. These weights are known as LPC coefficients^55^ (Fig. 1b). This results in the compression of the power spectrum of EEG into a few LPC coefficients and provides a highly efficient representation of the EEG signal. Our previous studies showed that EEG data represented in the high-dimensional space of these LPC coefficients manifest clear geometric separation for PD and control populations in the form of separate affine subspaces (a generalization of high-dimensional planes)^7,32^. An important advantage of LEAPD over other EEG analysis techniques is that it efficiently captures the unique spectral profile by compressing EEG oscillations into a few LPC parameters^31,32,56^ (Fig. 1c). To navigate LEAPD for capturing spectral profiles in a certain frequency band, EEG data are bandpass filtered before the calculation of LPC coefficients (Fig. 1d).

In this study, we utilized LEAPD to capture cognitive function by encoding EEG data using LPC and representing the encoded data in the LPC coefficient space. Although LEAPD was developed for binary classification, in this study our goal was to find an EEG-based marker that correlates with continuous cognitive scores. For this purpose, we utilized the geometric property of LPC coefficient space to compute our LEAPD index, where we correlate with clinical characteristics of PD by finding separate affine subspaces in the LPC coefficient space for cognitively impaired and cognitively normal participants (Fig. 1b). We hypothesized that after encoding EEG into vectors of LPC coefficients, there are two distinct affine subspaces—one for cognitive impairment and another for normal cognition and the relative distance from these affine subspaces (defined as the LEAPD index; Fig. 1b) highly correlates with cognitive function and can detect cognitive impairment. In summary, our goal was to generate an EEG-based cognitive index by capturing spectral changes in EEG with LPC (Fig. 1c) and then utilizing the geometric property of LPC coefficient space through LEAPD (Fig. 1b). Single channel LEAPD indices were calculated from individual electrodes and their geometric means provided a combined LEAPD index (Fig. 1d). LEAPD was originally designed to optimize its model parameters for binary classification by maximizing classifier accuracy as a cost function^7,32^. For this study, however, our main goal was to find an EEG-based cognitive index. Accordingly, we utilized Spearman’s rho correlation between LEAPD index and cognitive scores as the new cost function. This modification along with our sub-grouping of training data based on cognitive scores enabled us to utilize and direct LEAPD to identify specific spectral changes in EEG related primarily to cognitive function (as opposed to depression or PD diagnosis^7,32^) in a data-driven manner. We utilized EEG data from 149 participants (Table 1) and a single-round of 10-fold cross-validation for optimizing LEAPD using our new cost function. Once optimized, these parameters were kept unchanged for all analyses and performance evaluations across all participants except in robustness evaluation, where we varied the total number of electrodes. After optimizing the LEAPD parameters, we utilized the data from 149 participants (Table 1), applied several cross-validation schemes, and performed randomization tests by randomly shuffling cognitive scores among participants before using leave-one-out cross-validation to evaluate performance (Fig. 1e). Finally, we conducted a prospective out-of-sample test by calculating the LEAPD index for a separate dataset of 32 PD participants and compared its performance in terms of MoCA score correlation and detection of MoCA-based cognitive impairment (Fig. 1e, Table 1).

### LEAPD: theoretical framework for indexing cognition

The core aspect of LEAPD is encoding EEG data using LPC which rapidly encodes EEG time series by modeling each EEG data using preceding samples. The number of preceding samples used to model EEG data (the LPC order) dictates the level of detail captured for spectral profiles (total number of oscillations used for profiling spectral power). Fundamentally, LPC fits an autoregressive model to a time series. Suppose one has a time series with $N$ samples: $x\left(0\right),x\left(1\right),\ldots .x\left(N-1\right)$. Then, in $K$-th order LPC model, $x(n)$ is approximated by $n$ preceding data samples

1 $$x\left(n\right)=\epsilon \left(n\right)-\mathop{\sum }\limits_{i=1}^{K}{a}_{i}x\left(n-i\right)$$

where $${a}_{i}(i={1,2},\ldots K)$$ are the $$K$$ model parameters which are also known as LPC coefficients and$$\,\epsilon (n)$$ is the LPC model’s prediction error. Thus, given an EEG time series $$x\left(0\right),x\left(1\right),\ldots .x\left(N-1\right)$$ with *N* samples, LPC of order *K* generates $$K$$ number of LPC coefficients, ($${a}_{1},{a}_{2},..,{a}_{K})$$. These LPC coefficients (model parameters) represent a compressed encoding of the EEG time series. The LPC vectors were created using the LPC coefficients $$({\boldsymbol{a}}={\left[{a}_{1},{a}_{2}\ldots {a}_{K}\right]}^{T})$$ in the LEAPD approach. After representing EEG data in the multidimensional space of these model parameters (LPC coefficient space with $$K$$ dimensions), separate distinct affine subspaces (with $$<\, K$$ dimensions) are formed by data from cognitively impaired and cognitively normal participants. In our study, LEAPD is defined by the ratio of the distances from these affine subspaces such that it highly correlates with cognitive function and can detect cognitive impairment.

Principal component analysis (PCA) was used to identify these affine subspaces for each EEG electrode, and each single-electrode LEAPD index was calculated by taking the normalized ratio of distances from these affine subspaces. The number of principal components dictates the dimension of the affine subspaces. In particular, suppose the LPC order is *K* ($K$-dimensional LPC coefficient space) and the number of desired principal components is *n* (affine subspaces are *n*-dimensional; $n \,<\, K$). Assuming that there is a total $S$ number of participants with normal cognition (high cognitive scores) and with cognitive impairment (low cognitive scores) in the training dataset, the set for the vectors of LPC coefficients from *S* cognitively impaired subjects is ${V}_{1}$ with a dimension of $S\times K$. The ${i}^{{th}}$ row of$\,{V}_{1}$ is the LPC vector ${{\boldsymbol{a}}}_{{\boldsymbol{1}}{\boldsymbol{,}}{\boldsymbol{i}}}{\boldsymbol{;}}i\in \{\mathrm{1,2},..,S\}$ for ${i}^{{th}}$ subject with cognitive impairment. Similarly, let ${V}_{2}$ be the set for the vectors of LPC coefficients from $S$ subjects with normal cognition where the ${i}^{{th}}$ row of$\,{V}_{2}$ is the feature vector ${{\boldsymbol{a}}}_{{\boldsymbol{2}}{\boldsymbol{,}}{\boldsymbol{i}}}{\boldsymbol{;}}i\in \{\mathrm{1,2},..,S\}$ for the ${i}^{{th}}$ subject. Let ${{\boldsymbol{m}}}_{{\boldsymbol{1}}}\in {{\mathbb{R}}}^{1\times K}$ and ${{\boldsymbol{m}}}_{{\boldsymbol{2}}}\in {{\mathbb{R}}}^{1\times K}$ be the bias vectors of ${V}_{1}$ and ${V}_{2}$ respectively where each element ${m}_{c,i}$ is the mean of the ${i}^{{th}}$ column of ${V}_{c}$ where $c\in \{\mathrm{1,2}\}$. Now, let ${W}_{1}$ and ${W}_{2}$ be the scaled and unbiased form of ${V}_{1}$ and ${V}_{2}$. Therefore,

2 $${W}_{c}=\frac{{V}_{c}-{\left({D}_{c}\,Q\right)}^{T}}{\sqrt{S-1}}{\rm{;}}\,c\in \{1,2\}$$

3 $${D}_{c}=\left[\begin{array}{ccc}{m}_{c,1} & \cdots & 0\\ \vdots & \ddots & \vdots \\ 0 & \cdots & {m}_{c,K}\end{array}\right]{\rm{;}}\,c\in \left\{1,2\right\}$$

and $Q\in {{\mathbb{R}}}^{K\times S}$ with ${Q}_{{ij}}=1\forall i,j$. Finally, by performing singular value decomposition on ${W}_{1}$ and ${W}_{2}$ we get,

4 $${W}_{c}={U}_{c}{\Sigma }_{c}{P}_{c}^{T}\,{\rm{;}}\,c\in \left\{1,2\right\}$$

where the matrices ${U}_{c},{P}_{c}{;c}\in \{\mathrm{1,2}\}$ are left and right matrices with orthonormal basis vectors and ${\Sigma }_{1}$ and ${\Sigma }_{2}$ are diagonal matrices containing the singular values of ${W}_{1}$ and ${W}_{2}$. Thus, the basis vectors forming $n$-dimensional affine subspace for cognitive impairment are the first $n$ columns of ${P}_{1}$ corresponding to the $n$ most significant singular values. We denote these orthonormal vectors by ${{\boldsymbol{p}}}_{{\boldsymbol{1}}{\boldsymbol{,}}{\boldsymbol{1}}},{{\boldsymbol{p}}}_{{\boldsymbol{1}}{\boldsymbol{,}}{\boldsymbol{2}}},\cdots ,{{\boldsymbol{p}}}_{{\boldsymbol{1}}{\boldsymbol{,}}{\boldsymbol{n}}}$. Similarly, from ${P}_{2}$ we can obtain the $n$ orthonormal basis vectors for the affine subspace for normal cognition which are denoted by ${{\boldsymbol{p}}}_{{\boldsymbol{2}}{\boldsymbol{,}}{\boldsymbol{1}}},,{{\boldsymbol{p}}}_{{\boldsymbol{2}}{\boldsymbol{,}}{\boldsymbol{2}}},\cdots ,{{\boldsymbol{p}}}_{{\boldsymbol{2}}{\boldsymbol{,}}{\boldsymbol{n}}}$. Now, suppose the vector of LPC coefficients from a new participant is ${\boldsymbol{a}}$. Then, LEAPD determines the normalized distance ${D}_{1}$ and ${D}_{2}$ from point ${\boldsymbol{a}}$ to the respective affine subspaces for cognitive impairment and normal cognition. These distances are calculated by:

5 $${D}_{c}={{\bigg\Vert}{\boldsymbol{(}}{\boldsymbol{a}}-{{\boldsymbol{m}}}_{{\boldsymbol{c}}}{\boldsymbol{)}}-\mathop{\sum }\limits_{i=1}^{n}\frac{{{\boldsymbol{p}}}_{{\boldsymbol{c}}{\boldsymbol{,}}{\boldsymbol{i}}}^{T}{\boldsymbol{(}}{\boldsymbol{a}}-{{\boldsymbol{m}}}_{{\boldsymbol{c}}}{\boldsymbol{)}}}{{{\boldsymbol{p}}}_{{\boldsymbol{c}}{\boldsymbol{,}}{\boldsymbol{i}}}^{T}{{\boldsymbol{p}}}_{{\boldsymbol{c}}{\boldsymbol{,}}{\boldsymbol{i}}}}{{\boldsymbol{p}}}_{{\boldsymbol{c}}{\boldsymbol{,}}{\boldsymbol{i}}}{\bigg\Vert}}_{2}{\rm{;}}\,c\in \left\{1,2\right\}.$$

Finally, the LEAPD index ($\rho$) was calculated by taking the ratio of these normalized distances:

6 $$\rho =\frac{{D}_{2}}{{D}_{2}+{D}_{1}}.$$

LEAPD indices ($\rho$) ranged between 0 and 1, with high values corresponding to high cognitive scores. In particular, if $\rho < 0.5$, then the vector ${\boldsymbol{a}}$ is closer to the subspace with cognitive impairment, and when $\rho > 0.5$, it is closer to the subspace for normal cognition. We specified a LEAPD index value threshold of <0.5 to represent cognitive impairment which was determined principally by the relative distances from the affine subspaces of normal cognition and cognitive impairment. Before calculating the index, a zero phase, 6th order, Butterworth bandpass filter was applied to control the frequency region of the spectral profiling. After independently obtaining the single-electrode LEAPD index from multiple EEG electrodes, the indices were combined using their geometric mean to obtain a combined LEAPD index. In particular, after obtaining single-electrode LEAPD indices from $L$ electrodes ${\rho }_{i}{;i}\in \{\mathrm{1,2},\ldots ,L\}$, they were combined into a combined LEAPD index using geometric mean:

7 $${\rho }_{{combined}}={\left(\mathop{\prod }\limits_{i=1}^{L}{\rho }_{i}\right)}^{\frac{1}{L}}.$$

To optimize cognitively impaired and cognitively normal affine subspaces for achieving maximum correlation between LEAPD index and cognitive measures, we explored four key parameters of LEAPD: (1) frequency range of the bandpass filter between 2 and 34 Hz, (2) order of LPC between 2 and 10, (3) affine subspace dimensions, and (4) optimal EEG electrodes for the combined LEAPD index. The first three parameters were optimized for each EEG electrode by conducting an exhaustive search and maximizing Spearman’s rho correlation of the single-electrode LEAPD indices with cognitive scores using a single round of 10-fold cross-validation. After optimizing these three parameters for all EEG electrodes, we selected the optimal EEG electrodes based on their best individual performance during the exhaustive search. We utilized EEG data from 149 participants (Table 1) for these optimizations. Once these parameters were determined, we kept them unchanged for all analyses and performance evaluations across all participants except the robustness performance, where we varied the total number of electrodes.

Similarly to the MoCA score, utilizing LEAPD for cognitive tests from NIH-Toolbox was achieved by identifying affine subspaces for high cognitive scores and low cognitive scores. For each of the cognitive tests from NIH-Toolbox, the median of the cognitive scores across all participants was used as a cutoff to divide participants into high and low-score cognitive groups.

### Performance evaluation

We calculated spectral power in the delta (1−4 Hz), theta (4−8 Hz), alpha (8−13 Hz), beta (13−30 Hz), and gamma (31−100 Hz) bands for our traditional EEG spectral analyses using the EEG data from 149 participants (Table 1). The spectral powers were calculated for each participant using all individual EEG electrodes. There were 300 spectral features for each participant and 44,700 measurements from the whole dataset, with the dimensions of 5 bands × 149 participants × 60 electrodes. We also calculated the log-spectral ratio of alpha and theta band power. We calculated age-adjusted partial Spearman’s rho correlations between cognitive scores and these spectral features which we compared with the correlation between the cognitive score and the combined LEAPD index.

LEAPD performance was quantified using non-parametric Age-adjusted Spearman’s rho partial correlation. To further explore LEAPD’s relationship with MoCA, we used regression analysis, in which we considered linear and quadratic models. We inspected 4 metrics during regression: *R*^2^; root-mean-square error of the combined LEAPD index (RMSE); F-test on the regression model (F-statistic) and Akaike information criterion (AIC). To quantify classification results, we used sensitivity, specificity, classifier accuracy rate, area under the receiver operating characteristic curve (AUC), positive predictive value (PPV), negative predictive value (NPV), and odds ratio. To validate our results, we utilized EEG data from 149 participants (Table 1) and performed leave-one-out cross-validations using all participants with 1) their true cognitive scores and 2) after randomly shuffling cognitive scores among participants (randomization test). We also performed 5-fold and 10-fold cross-validations in 100 independent rounds of data shuffling. Finally, we conducted an external validation of the LEAPD performance for MoCA score with a prospective out-of-sample test dataset of 32 PD patients (Table 1). LEAPD parameters derived from our dataset of 149 participants in Table 1 were kept unchanged. In the testing phase, we calculated the LEAPD index for the 32 new and separately collected test PD participants and evaluated the performance in correlation with MoCA scores and MoCA-based cognitive impairment classification.

To investigate how the total number of selected EEG electrodes for the combined LEAPD index influenced performance, we utilized EEG data from 149 participants (Table 1), performed the leave-one-out cross-validation, and calculated Spearman’s rho correlation coefficient, classifier accuracy rate, and AUC. The electrodes were selected based on the order (high to low) of their individual single-electrode LEAPD performances of MoCA correlations (Fig. 2c). For investigating the robustness of the LEAPD approach in truncation analysis, we started with the full EEG dataset of 149 participants (average EEG length: 2.7 min; *n* = 149; Table 1), gradually truncated the dataset up to 10% with 5% increments, and quantified performance for each case using leave-one-out cross-validation. Truncation was applied to the EEG data from all 149 participants (Table 1) starting from the end of the EEG recordings. We repeated the same procedure to the MoCA-shuffled dataset and compared the performance.

### Statistical analysis

As the distribution of the bounded LEAPD index is non-gaussian, we utilized the Spearman (rank) partial correlation method (‘partialcorr’ function in Matlab and ‘pcor’ command in R) controlling for the participants’ age to measure correlation while accounting for group-level age differences (Table 1)^34,35^. Two-sided non-parametric Wilcoxon rank-sum tests were performed to measure group-level differences. We included potentially confounding factors such as sex, L-dopa equivalent daily dose (LEDD), age, disease duration, Unified Parkinson’s Disease Rating Scale part III (UPDRS III), and patients’ geriatric depression scale (GDS) by incorporating these variables in a linear model (*lm* in R) along with MoCA scores and LEAPD indices obtained through leave-one-out cross-validation. We reported F-statistics from Analyses of variance (ANOVA; *anova* in R) and the effect sizes were calculated via partial eta squared (*etaSquared* in R). Sensitivity analyses were further conducted using Spearman partial correlation (*pcor.test* in R) between LEAPD and MoCA, controlling for sex, age, and GDS in all participants, as well as LEDD, UPDRS III, and disease duration in PD patients. We also tested whether PD had any statistically significant impact on the correlation between the combined LEAPD index and MoCA score using linear mixed-effect models. To compare regression models, we used the likelihood ratio test. All statistical procedures were performed using MATLAB 2021b and R (version 4.1.2). All data and statistical analyses were reviewed by the Biostatistics and Research Design Core in the Institute for Clinical and Translational Science at the University of Iowa.

### Reporting summary

Further information on research design is available in the Nature Research Reporting Summary linked to this article.

### Supplementary information

SUPPLEMENTARY MATERIALS

Reporting Summary

Supplementary Video 1

## Data Availability

The datasets generated and/or analyzed during the current study are available at: http://narayanan.lab.uiowa.edu and at https://openneuro.org/datasets/ds004584.

## Code availability

The underlying codes generated and utilized for this study are available at https://github.com/MDFahimAnjum and http://narayanan.lab.uiowa.edu.
