# Supplementary material for: Resting-state EEG measures cognitive impairment in Parkinson’s disease
Source: NPJ Parkinsons Dis. 2024 Jan 3;10:6. doi: 10.1038/s41531-023-00602-0 (PMC10764756; doi:10.1038/s41531-023-00602-0)
Supplement: Supplementary file 1 — SUPPLEMENTARY MATERIALS [file 41531_2023_602_MOESM1_ESM.pdf]

SUPPLEMENTARY MATERIALS FOR

Resting-state EEG Measures Cognitive Impairment in Parkinson's Disease

Anjum et al.

Supplementary Table 1: Performance summary of the LEAPD in 5-fold and 10-fold cross-validation

		100 x 5-fold CV			100 x 10-fold CV		
		All participants	Parkinson's disease only	Controls only	All participants	Parkinson's disease only	Controls only
Spearman's-rho correlation with cognitive measures	MoCA	0.67***	0.64***	0.46***	0.68***	0.66***	0.46***
	NIH PVT	0.63***	0.68***	0.51***	0.64***	0.69***	0.52***
	NIH PCPST	0.58***	0.57***	0.09***	0.58***	0.57***	0.11***
	NIH DCCST	0.66***	0.66***	0.64***	0.68***	0.69***	0.67***
	NIH FICAT	0.60***	0.59***	0.46***	0.61***	0.60***	0.47***
	NIH PSMT	0.55***	0.53***	0.03**	0.56***	0.54***	0.03**
Linear regression model for MoCA	R^2	0.37	0.34	0.31	0.38	0.35	0.32
	RMSE	0.12	0.12	0.10	0.12	0.12	0.10
	F-statistic	8800.46***	5043.47***	2175.08***	9315.00***	5361.56***	2348.82***
	AIC	-21768.18	-14125.02	-8799.03	-21829.22	-14231.11	-8802.15
Quadratic regression model for MoCA	R^2	0.44	0.42	0.34	0.45	0.44	0.36
	RMSE	0.11	0.11	0.10	0.11	0.11	0.10
	F-statistic	5828.74***	3654.98***	1284.82***	6202.83***	3899.21***	1383.83***
	AIC	-23462.63	-15527.00	-9063.35	-23617.64	-15702.14	-9075.83
Detection of MoCA-based cognitive impairment	Accuracy %	80.50	78.88	83.80	80.97	78.82	85.35
	AUC	0.89	0.89	0.83	0.90	0.89	0.84
	Sensitivity %	78.80	82.00	63.36	80.06	82.85	66.64
	Specificity %	81.78	75.36	89.71	81.65	74.28	90.76
	PPV %	76.50	78.96	64.06	76.66	78.41	67.62
	NPV %	83.67	78.78	89.43	84.47	79.34	90.38
	Odds ratio	16.68	13.93	15.06	17.86	13.94	19.60

Abbreviations: CV = Cross-validation; RMSE = root mean squared error; PPV = Positive predictive value; NPV = Negative Predictive value; AIC = Akaike information criterion; AUC = Area under the receiver operating characteristic curve; MoCA = Montreal Cognitive Assessment; NIH = National Institutes of Health; PVT = Picture vocabulary test; FICAT = Flanker inhibitory control and attention test; DCCST = Dimensional change card sorting test; PCPST = Pattern comparison processing speed test; PSMT = Picture sequence memory test. All cross-validations were repeated 100 times with data shuffling and results were averaged. For the regression models, *p* values show the statistical significance of the predictor variable (combined LEAPD index). In all cases, *p* value <0.001***, <0.01**. Data from 149 participants (Table 1).

Supplementary Table 2: LEAPD performance for leave-one-out cross-validation after shuffling scores of cognitive measures

		All participants (n=149)	PD only (n=100)	Controls only (n=49)
Spearman's-rho correlation with cognitive measures	MoCA	0.02	0.05	-0.07
	NIH PVT	0.06	0.12	-0.05
	NIH PCPST	-0.1	-0.12	-0.09
	NIH DCCST	-0.05	-0.01	-0.07
	NIH FICAT	0.08	0.21*	-0.11
	NIH PSMT	0.04	0.09	-0.06
Linear regression model for MoCA	R^2	0.01	0.02	<0.01
	RMSE	0.09	0.09	0.08
	F-statistic	1.40	1.63	0.01
	AIC	-293.35	-189.11	-103.08
	Log-likelihood	149.68	97.56	54.54
Quadratic regression model for MoCA	R^2	0.02	0.03	0.01
	RMSE	0.09	0.09	0.08
	F-statistic	1.16	1.26	0.21
	AIC	-292.29	-188.02	-102.51
	Log-likelihood	150.14	98.01	54.75
Detection of MoCA-based cognitive impairment	Accuracy %	50.34	50.00	51.02
	AUC	0.49	0.51	0.51
	Sensitivity %	54.69	50.00	63.64
	Specificity %	47.06	50.00	40.74
	PPV %	43.75	42.00	46.67
	NPV %	57.97	58.00	57.89
	Odds ratio	1.07	1.00	1.20

Abbreviations: RMSE = root mean squared error; AUC = Area under the receiver operating characteristic curve; AIC = Akaike information criterion; PPV = positive predictive value; NPV = negative predictive value; MoCA = Montreal Cognitive Assessment; NIH = National Institutes of Health; PVT = Picture vocabulary test; FICAT = Flanker inhibitory control and attention test; DCCST = Dimensional change card sorting test; PCPST = Pattern comparison processing speed test; PSMT = Picture sequence memory test. Statistical test *p* value <0.05*. Data from 149 participants (Table 1).

Supplementary Figure 1

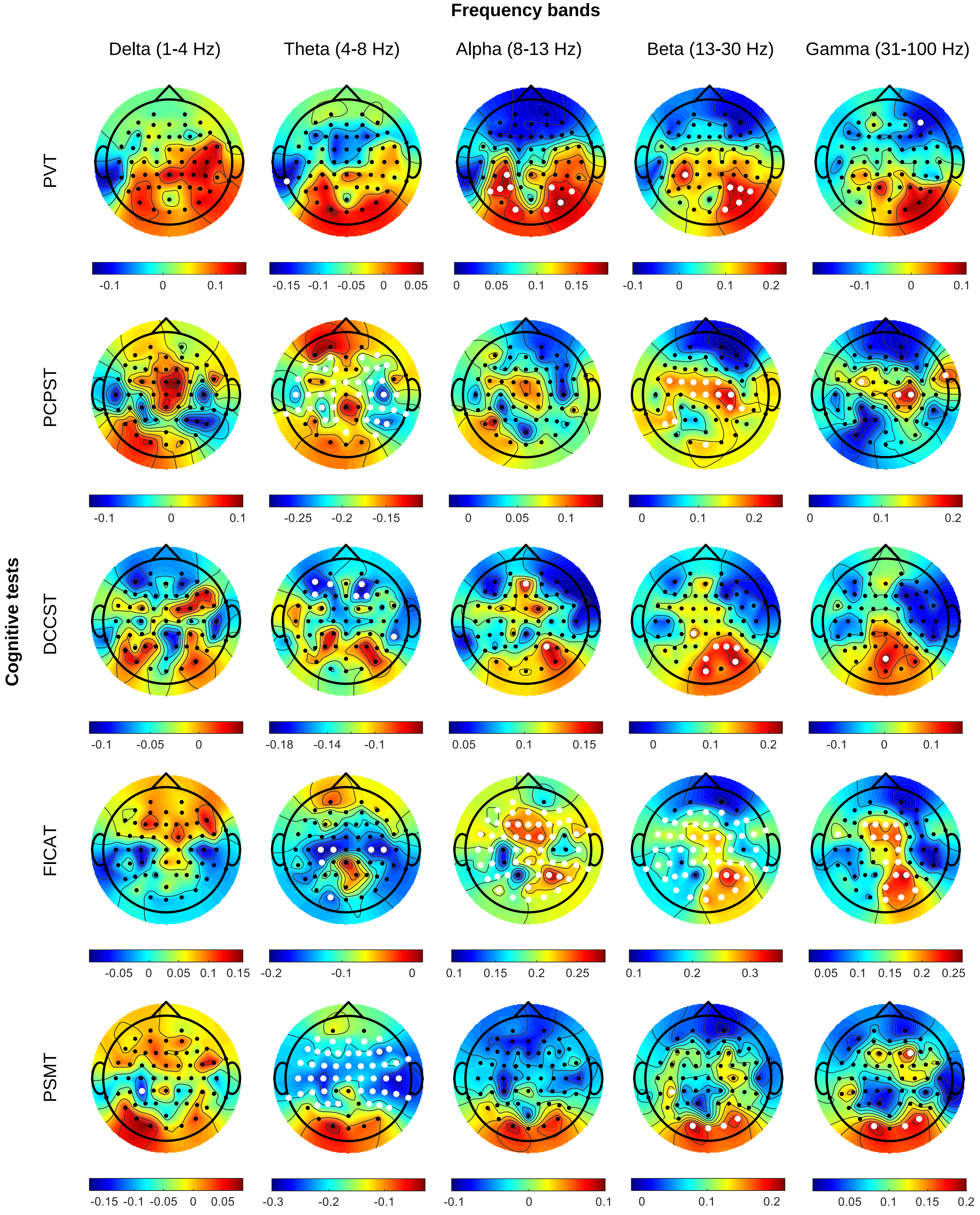

Correlation between EEG spectral powers and cognitive tests from NIH-Toolbox. Topographic plots show age-adjusted Spearman's rho correlation between delta, theta, alpha, beta and gamma power of EEG and five cognitive tests from NIH-Toolbox.

Electrodes with statistically significant correlations (p-value< 0.05) are marked with white dots. PVT = Picture vocabulary test; FICAT = Flanker inhibitory control and attention test; DCCST = Dimensional change card sorting test; PCPST = Pattern comparison processing speed test; PSMT = Picture sequence memory test. Data from 149 participants (Table 1).

Supplementary Figure 2

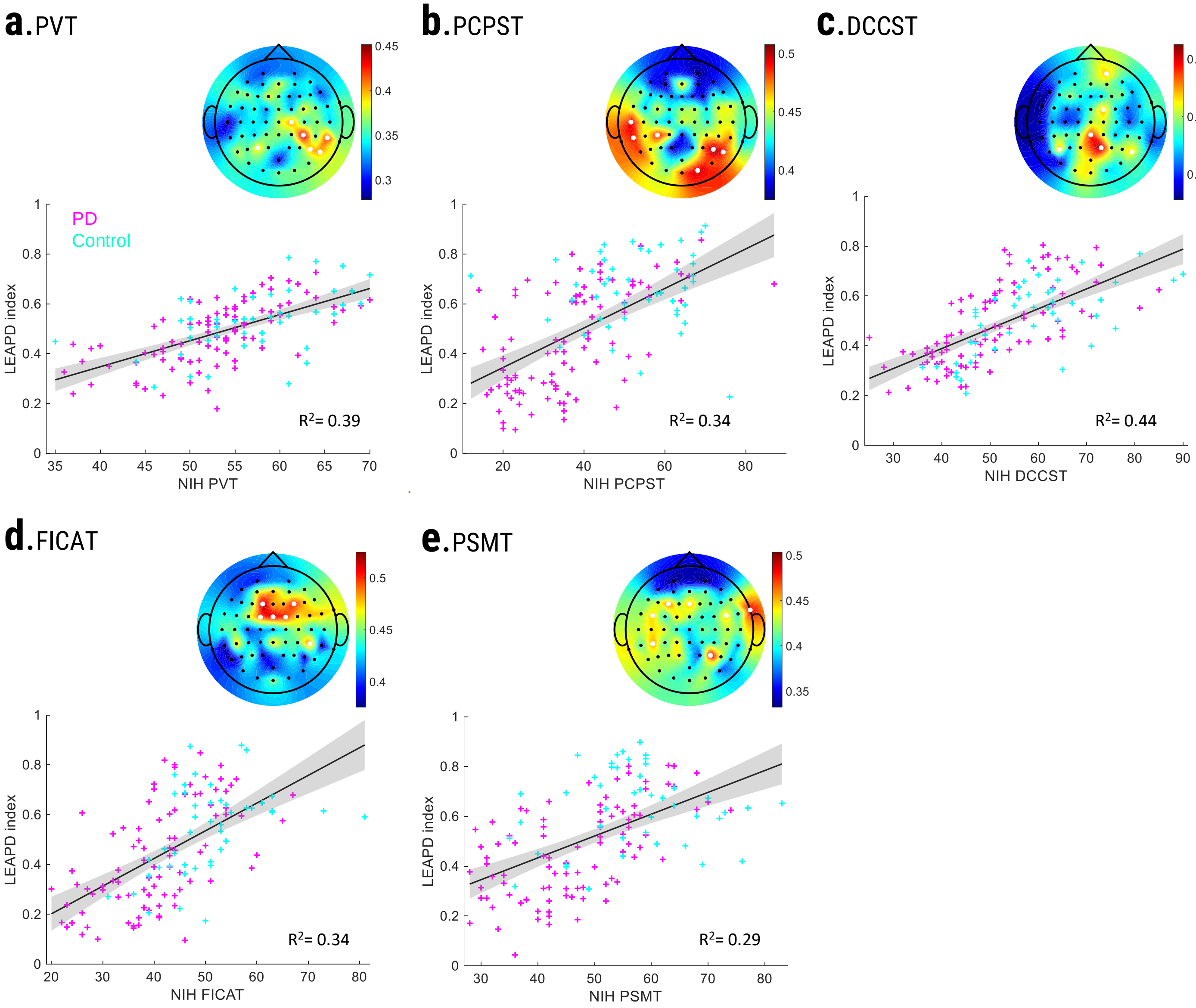

Performance of LEAPD for cognitive tests from NIH-Toolbox in leave-one-out cross-validation. Scatter plots for the linear regression models between the cognitive test scores and LEAPD indices for all participants (Table 1, n=149) with (pink) and without PD (cyan). The solid line shows a linear fit to the data with 95% confidence intervals (shaded regions). LEAPD was trained individually for each case (panel a-e) and hyperparameters were optimized. The topographic plots show correlations between single-electrode LEAPD indices and cognitive test scores during optimal parameter selection and the selected electrodes (marked as white dots) for generating the combined LEAPD index for each case (panel a-e). PD = Parkinson's disease, PVT = Picture vocabulary test; FICAT = Flanker inhibitory control and attention test; DCCST = Dimensional change card sorting test; PCPST = Pattern comparison processing speed test; PSMT = Picture sequence memory test.

Supplementary Figure 3

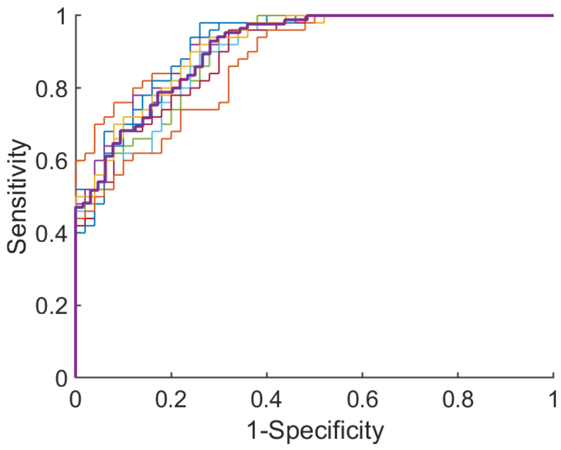

Performance of LEAPD in balanced groups during leave-one-out cross-validation: Light lines (n=10) show receiver operative characteristic (ROC) curves of LEAPD after randomly sub-sampling the dataset with 50 cognitively impaired and 50 cognitively normal participants after leave-one-out cross-validation. The thick line shows ROC curve for the dataset of 149 participants (Table 1).

Supplementary Video 1

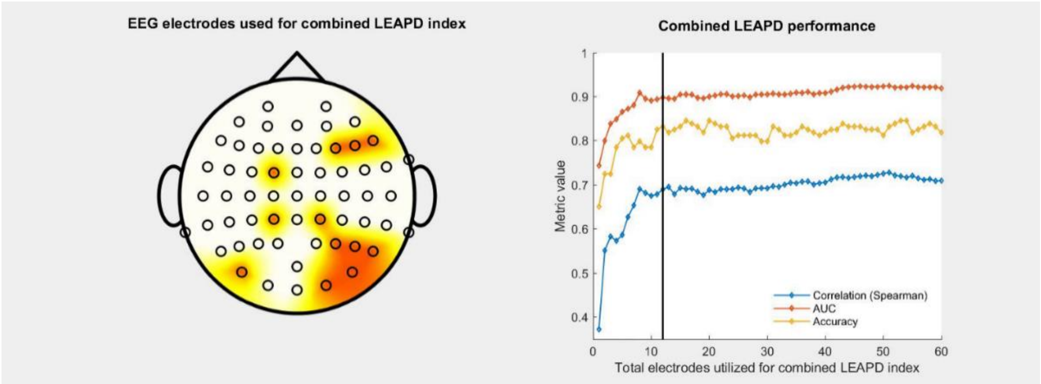

Performance of LEAPD with varying EEG electrodes. Effect of the total number of selected EEG electrodes for the combined LEAPD index on performance during leave-one-out cross-validations with 149 participants (Table 1). The topographical plot on the ***left* panel** highlights the selected EEG electrodes utilized for generating the combined LEAPD index. Selected EEG electrodes are marked in orange. The order of the selected electrodes was determined by their individual performances of the single-electrode LEAPD index in 10-fold cross-validation (Fig. 2c). Concurrently, the ***right* panel** shows the performance of the combined LEAPD index (generated by the selected EEG electrodes) in Spearman's rho correlation with MoCA scores (*blue*), classifier accuracy (*yellow*), and AUC (*red*). The black vertical marker shows the current performance corresponding to the left panel. Abbreviation: AUC = Area under the receiver operating characteristic curve.
