# Supplementary material for: Resting-state EEG measures cognitive impairment in Parkinson’s disease
Source: NPJ Parkinsons Dis. 2024 Jan 3;10:6. doi: 10.1038/s41531-023-00602-0 (PMC10764756; doi:10.1038/s41531-023-00602-0)
Supplement: Supplementary file 2 — Reporting Summary [file 41531_2023_602_MOESM2_ESM.pdf]

nature portfolio

Corresponding author(s): Md Fahim Anjum

Last updated by author(s): Oct 30, 2023

# Reporting Summary

Nature Portfolio wishes to improve the reproducibility of the work that we publish. This form provides structure for consistency and transparency in reporting. For further information on Nature Portfolio policies, see our Editorial Policies and the Editorial Policy Checklist.

## Statistics

For all statistical analyses, confirm that the following items are present in the figure legend, table legend, main text, or Methods section.

| n/a | Confirmed | |
|---|---|---|
| ☐ | ☒ | The exact sample size (*n*) for each experimental group/condition, given as a discrete number and unit of measurement |
| ☐ | ☒ | A statement on whether measurements were taken from distinct samples or whether the same sample was measured repeatedly |
| ☐ | ☒ | The statistical test(s) used AND whether they are one- or two-sided<br>*Only common tests should be described solely by name; describe more complex techniques in the Methods section.* |
| ☐ | ☒ | A description of all covariates tested |
| ☐ | ☒ | A description of any assumptions or corrections, such as tests of normality and adjustment for multiple comparisons |
| ☐ | ☒ | A full description of the statistical parameters including central tendency (e.g. means) or other basic estimates (e.g. regression coefficient) AND variation (e.g. standard deviation) or associated estimates of uncertainty (e.g. confidence intervals) |
| ☐ | ☒ | For null hypothesis testing, the test statistic (e.g. *F*, *t*, *r*) with confidence intervals, effect sizes, degrees of freedom and *P* value noted<br>*Give P values as exact values whenever suitable.* |
| ☒ | ☐ | For Bayesian analysis, information on the choice of priors and Markov chain Monte Carlo settings |
| ☐ | ☒ | For hierarchical and complex designs, identification of the appropriate level for tests and full reporting of outcomes |
| ☐ | ☒ | Estimates of effect sizes (e.g. Cohen's *d*, Pearson's *r*), indicating how they were calculated |

*Our web collection on statistics for biologists contains articles on many of the points above.*

## Software and code

Policy information about availability of computer code

| | |
|---|---|
| Data collection | Resting-state EEG was collected from participants while they sat in a quiet room with their eyes open for a few minutes using a 64-channel actiCAP and Brain Vision system (Brain Products GmbH) with a 0.1-Hz high pass filter and a sampling frequency of 500 Hz. |
| Data analysis | All statistical procedures and data analyses were performed using MATLAB 2021b and R (version 4.1.2). |

For manuscripts utilizing custom algorithms or software that are central to the research but not yet described in published literature, software must be made available to editors and reviewers. We strongly encourage code deposition in a community repository (e.g. GitHub). See the Nature Portfolio guidelines for submitting code & software for further information.

## Data

Policy information about availability of data

All manuscripts must include a data availability statement. This statement should provide the following information, where applicable:

- Accession codes, unique identifiers, or web links for publicly available datasets
- A description of any restrictions on data availability
- For clinical datasets or third party data, please ensure that the statement adheres to our policy

The datasets and the underlying codes generated and/or analyzed during the current study are available at: http://narayanan.lab.uiowa.edu and at https://openneuro.org/datasets/ds004584

## Human research participants

Policy information about studies involving human research participants and Sex and Gender in Research.

| | |
|---|---|
| Reporting on sex and gender | Sex of the participants were determined by self-reports. All participants provided written consent for obtaining and sharing individual-level data. Findings in this study applies to both sex. The study was designed and data were collected from both sex. The initial dataset (n=149) contains 68 male and 32 female participants with Parkinson's disease (PD). It also contains 26 male and 23 female control participants without PD. Sex-based analyses were performed by including sex as a potential confounding factor in statistical models and by controlling for sex in Spearman partial correlations. The out-of-sample dataset (n=32) contains 21 male and 11 female participants with PD. |
| Population characteristics | In the initial dataset (n=149), average age of PD patients (n=100) was 68.53 ± 8.06 years and it was 70.91 ± 7.62 years for control participants (n=49). Years since PD diagnosis was 4.64 ± 3.79 years. In the out-of-sample dataset (n=32) of 32 PD patients, average age was 67.7 ± 7.9 years. Duration since PD diagnosis was 3.8 ± 3.5 years. |
| Recruitment | We recruited 100 PD participants from the Movement Disorders Clinic at the University of Iowa, Iowa City between 2017 and 2022. Additionally, 49 demographically similar controls without known neurological disease were recruited from the general Iowa City community between 2017 and 2022 through the Seniors Together in Aging Research registry (https://icts.uiowa.edu/star). We also recruited 32 new and independent PD participants from the Aerobic Exercise in Parkinson's Disease (NCT03808675) for our prospective out-of-sample test. There were no self-selection or other kinds of bias in the recruitment. |
| Ethics oversight | The study was approved by the University of Iowa Institutional Review Board (protocol # 201707828). Written informed consent was provided by all participants. |

Note that full information on the approval of the study protocol must also be provided in the manuscript.

# Field-specific reporting

Please select the one below that is the best fit for your research. If you are not sure, read the appropriate sections before making your selection.

☒ Life sciences ☐ Behavioural & social sciences ☐ Ecological, evolutionary & environmental sciences

For a reference copy of the document with all sections, see nature.com/documents/nr-reporting-summary-flat.pdf

# Life sciences study design

All studies must disclose on these points even when the disclosure is negative.

| | |
|---|---|
| Sample size | We recruited 100 PD participants and 49 demographically similar controls without known neurological disease. We also recruited 32 independent PD participants from the Aerobic Exercise in Parkinson's Disease (NCT03808675) for out-of-sample test. No sample size calculation was performed. The sample size of our study is one of the largest among other studies on the literature. |
| Data exclusions | No data were excluded from the analysis. |
| Replication | The study utilized cross-validation schemes with the initial dataset of 149 participants (100 PD, 49 control) where subject-level cross-validation technique was implemented with various values in k-folds (k=5,10 and leave-one-out). Performance were averaged across the cross-validation schemes. For investigating the robustness of the LEAPD approach in truncation analysis, we started with the full EEG dataset (average EEG length: 2.7 min; n=149), gradually truncated the dataset up to 10% with 5% increments, and quantified performance for each case using leave-one-out cross-validation. Truncation was applied to the EEG data from all participants (n=149) starting from the end of EEG recordings. We repeated the same procedure to the MoCA-shuffled dataset and compared the performance. For investigating how the total number of selected EEG electrodes for the combined LEAPD index influenced performance, we utilized leave-one-out cross-validation and calculated Spearman's rho correlation coefficient, classifier accuracy rate, and AUC. We utilized the Spearman (rank) partial correlation method controlling for the participants' age to measure correlation while accounting for group-level age differences. We included potentially confounding factors such as sex, L-dopa equivalent daily dose (LEDD), age, disease duration, Unified Parkinson's Disease Rating Scale part III (UPDRS III), and patients' geriatric depression scale (GDS) by incorporating these variables in a linear model along with MoCA scores and LEAPD indices obtained through leave-one-out cross-validation. Spearman partial correlation between LEAPD and MoCA, controlling for sex, age, and GDS in all participants, as well as LEDD, UPDRS III, and disease duration in PD patients. We checked the potential effects of the group sizes by sub-sampling the dataset with equal numbers of participants for each group in leave-one-out cross-validation. The replication methods were successful. Finally, we conducted an external validation of the LEAPD performance for MoCA score with the out-of-sample test dataset of 32 PD patients. LEAPD parameters derived from our initial dataset of 149 participants were kept unchanged. In the out-of-sample test, we calculated the LEAPD index for the 32 new and separately collected test PD participants and evaluated the performance in correlation with MoCA scores and MoCA-based cognitive impairment classification. |
| Randomization | In the study, data were analyzed with randomly shuffled cognitive scores among subjects which showed no statistically significant correlation with cognitive scores or detection of cognitive impairment suggesting that the correlations with cognitive measures are not achieved through fitting noise or data artifacts. During the cross-validations (CVs), each k-fold CV was repeated 100 times with independent shuffling of the folds. We analyzed the performance after randomly sub-sampling the dataset (n=10) with 50 cognitively impaired and 50 cognitively normal |

participants after leave-one-out cross-validation.

Blinding

Investigator who developed methodology, evaluated the results, measured performance and conducted data analysis and statistical tests were blinded to group allocation during data collection.

# Reporting for specific materials, systems and methods

We require information from authors about some types of materials, experimental systems and methods used in many studies. Here, indicate whether each material, system or method listed is relevant to your study. If you are not sure if a list item applies to your research, read the appropriate section before selecting a response.

## Materials & experimental systems

| n/a | Involved in the study |
|---|---|
| ☒ | ☐ Antibodies |
| ☒ | ☐ Eukaryotic cell lines |
| ☒ | ☐ Palaeontology and archaeology |
| ☒ | ☐ Animals and other organisms |
| ☒ | ☐ Clinical data |
| ☒ | ☐ Dual use research of concern |

## Methods

| n/a | Involved in the study |
|---|---|
| ☒ | ☐ ChIP-seq |
| ☒ | ☐ Flow cytometry |
| ☒ | ☐ MRI-based neuroimaging |
